# Supplementary material for: Iterative improvement in the automatic modular design of robot swarms
Source: PeerJ Comput Sci. 2020 Dec 7;6:e322. doi: 10.7717/peerj-cs.322 (PMC7924708; doi:10.7717/peerj-cs.322)
Supplement: Supplemental Information 3 [file peerj-cs-06-322-s003.zip › argos3/doc/api/standalone/a00451_source.html]

ARGoS: plugins/robots/foot-bot/control\_interface/ci\_footbot\_turret\_actuator.h Source File


- Main Page
- Related Pages
- Namespaces
- Classes
- Files

- File List
- File Members

# plugins/robots/foot-bot/control\_interface/ci\_footbot\_turret\_actuator.h

Go to the documentation of this file.

```
00001 
00043 #ifndef CCI_FOOTBOT_TURRET_ACTUATOR_H
00044 #define CCI_FOOTBOT_TURRET_ACTUATOR_H
00045 
00046 namespace argos {
00047    class CCI_FootBotTurretActuator;
00048 }
00049 
00050 #include <argos3/core/control_interface/ci_actuator.h>
00051 #include <argos3/core/utility/math/angles.h>
00052 
00053 namespace argos {
00054 
00055    class CCI_FootBotTurretActuator : virtual public CCI_Actuator {
00056 
00057    public:
00058 
00060       enum ETurretModes {
00061          MODE_OFF,
00062          MODE_PASSIVE,
00063          MODE_SPEED_CONTROL,
00064          MODE_POSITION_CONTROL
00065       };
00066 
00067    public:
00068 
00069       static const CRange<SInt32> SPEED_RANGE;
00070       static const CRange<Real>   NORMALIZED_SPEED_RANGE;
00071 
00072    public:
00073 
00077       virtual ~CCI_FootBotTurretActuator() {}
00078 
00089       virtual void SetRotation(const CRadians& c_angle) = 0;
00090 
00101       virtual void SetRotationSpeed(SInt32 n_speed_pulses) = 0;
00102 
00108       virtual void SetMode(ETurretModes e_mode) = 0;
00109 
00118       void SetActiveWithRotation(const CRadians& c_angle);
00119 
00124       void SetSpeedControlMode();
00125 
00130       void SetPositionControlMode();
00131 
00136       void SetPassiveMode();
00137 
00138 #ifdef ARGOS_WITH_LUA
00139       virtual void CreateLuaState(lua_State* pt_lua_state);
00140 #endif
00141    };
00142 
00143 }
00144 
00145 #endif
```

---

Generated on 10 Jul 2018 for ARGoS by 
 1.6.1
